# iDualG4: A Dual-Channel Deep Learning Framework for Predicting In Vivo G-Quadruplexes

**DOI:** 10.3390/biom16050693

**Published:** 2026-05-07

**Authors:** Haitao Li, Li Dong, Yue Jia, Chunhou Zheng, Pijing Wei

**Affiliations:** 1 Key Laboratory of Intelligent Computing and Signal Processing, School of Artificial Intelligence, Anhui University, 111 Jiulong Road, Hefei 230601, China; liht@ahu.edu.cn (H.L.); zhengch99@126.com (C.Z.); 2 Physical Science and Information Technology, Anhui University, 111 Jiulong Road, Hefei 230601, China; 19840069970@163.com (L.D.); jy03207790@163.com (Y.J.)

**Keywords:** dual-channel network, G-quadruplex, interpretability, epigenetic regulation

## Abstract

G-quadruplexes (G4s) are non-canonical nucleic acid secondary structures that help maintain genomic stability and regulate gene transcription. Although the genome contains a vast number of putative G4-forming sequences (PQSs, sequences with intrinsic in vitro G4-forming potential), only a small fraction fold stably into G4 structures within the complex chromatin environment of living cells. Existing deep learning approaches improve predictive accuracy by incorporating cell line–specific epigenetic data; however, their heavy reliance on costly, large-scale sequencing assays (e.g., ChIP-seq) limits broader application to clinical samples and newly profiled cell lines. To address this challenge, we propose iDualG4, an interpretable dual-channel deep learning framework that uses DNA sequence as the only input at inference time. By leveraging a pretrained Enformer module, iDualG4 infers epigenomic proxy features directly from DNA sequence and integrates them with local sequence features, thereby replacing the need for newly measured cell-specific epigenomic assays during prediction. Evaluations across multiple cell lines, including K562, demonstrate that iDualG4 significantly outperforms existing methods, particularly in handling imbalanced data (achieving an AUPR of 0.981 on K562). Interpretability analysis based on DeepSHAP indicates that iDualG4 provides an in vivo G4 prediction tool combining high precision and interpretability without the need for additional experimental sequencing data, and offers a novel computational framework for elucidating how sequence and the epigenetic environment jointly determine genomic G4 formation.

## 1. Introduction

Non-canonical nucleic acid secondary structures are essential regulators of genomic spatiotemporal organization. Among these, G-quadruplexes (G4s)—four-stranded helical structures formed by guanine-rich sequences via Hoogsteen hydrogen bonding [1]—are evolutionarily conserved and widely distributed in functional genomic regions such as promoters, telomeres, and 5′ UTRs. Extensive research has established that G4s function as dynamic epigenetic switches, participating in transcriptional regulation, DNA replication initiation, translational control, and maintenance of genomic stability [2,3,4].

Despite the substantial evidence supporting their regulatory significance, accurately identifying in vivo G4s that stably fold within the chromatin context at a genomic scale remains a major challenge. In recent years, high-throughput sequencing technologies such as G4-seq [5], G4 ChIP-seq [6], and G4 CUT&Tag [7] have revealed a critical discrepancy between putative G4-forming sequences (PQS) encoded in the genome and their actual folding state within living cells. For instance, while G4-seq data indicates that the human genome contains over 700,000 PQSs [5], in vivo profiling by Hänsel-Hertsch et al. (2016) detected only approximately 10,000 significant G4 peaks using ChIP-seq [6]. Consequently, detectable stable in vivo G4 signals correspond to only a small fraction (approximately 1–2%) of computationally predicted PQSs. This disparity suggests that relying solely on sequence motifs or in vitro folding potential introduces a high rate of false positives, as physiological folding is heavily constrained by the cellular environment. Although methods like G4 ChIP-seq and G4 CUT&Tag capture in vivo G4s with improved specificity [6,7], they are limited by workflow complexity, antibody dependence, variable signal-to-noise ratios, and low sample scalability, making them unsuitable for systematic screening across multiple cell lines or clinical tissues.

To address these limitations, computational prediction methods have evolved from “rule-based” approaches to “multi-modal learning” frameworks that fuse diverse omics data. Early tools, such as Quadparser, relied solely on regular expressions to screen for PQSs, a strategy prone to high false positive rates. With the advent of artificial intelligence, machine learning models like XGBoost [8] significantly improved performance by learning features such as base composition and sequence complexity; however, they struggled to characterize the impact of the chromatin environment on G4 folding. More recent methods have explicitly integrated cell-specific epigenetic information. For example, G4Beacon [9] combines in vitro G4 sequences with ATAC-seq signals in a gradient boosting framework to enhance accuracy, while epiG4NN [10] utilizes a ResNet architecture to integrate DNA sequences with histone modifications (e.g., H3K4me3 and H3K27ac), achieving cell-line-specific predictions. In parallel, advances in G4-related predictive modeling, such as G4STAB [11], have focused on sequence-based architectures for predicting G4 stability, reflecting continued development of computational tools in this area. Notably, these studies confirmed a strong correlation between marks like H3K4me3 and G4 formation [10], demonstrating that incorporating cell-specific epigenetic features is an effective pathway to enhance in vivo prediction.

Despite the improved performance reported on specific benchmark datasets, the broader applicability of current G4 prediction models remains limited by three key bottlenecks. First, many state-of-the-art methods exhibit a strong dependence on matched experimental epigenomic profiles for the target cell line during inference; for example, models such as G4Beacon and epiG4NN typically require accompanying sequencing data (e.g., ChIP-seq or ATAC-seq), which are often unavailable for clinical specimens or newly studied cell lines, thereby substantially constraining generalization. Second, most existing approaches adopt relatively narrow input windows (approximately 100–200 bp), and this limited receptive field may fail to fully exploit long-range regulatory cues embedded in sequence contexts spanning thousands of bases, ultimately weakening the characterization of the broader chromatin background [12]. To address this challenge, foundation-model-based genomics has recently attracted increasing attention. Studies such as the systematic benchmarking of DNA foundation models for genomic tasks [13], ChromBERT for modeling chromatin-related features [14], and the generalized transcription foundation model GET [15] show that large-scale sequence pretraining can help capture regulatory grammar and long-range dependencies across genomic regions.

Third, despite their predictive advantages, deep learning–based predictors are frequently criticized for limited interpretability, as they tend to operate as “black boxes” that hinder quantitative attribution of G4 folding stability to specific sequence motifs or epigenetic determinants [16]. Addressing this ‘black box’ issue, recent studies in interpretable sequence modeling have explored how regulatory syntax can be extracted from trained deep neural networks. For instance, benchmarks of sequence-function interpretability methods [17] and frameworks identifying cell-type-specific sequence rules, such as those related to Hippo signaling [18], suggest that interpretability techniques can provide biologically meaningful insights into motif interactions.

**Figure 1:**
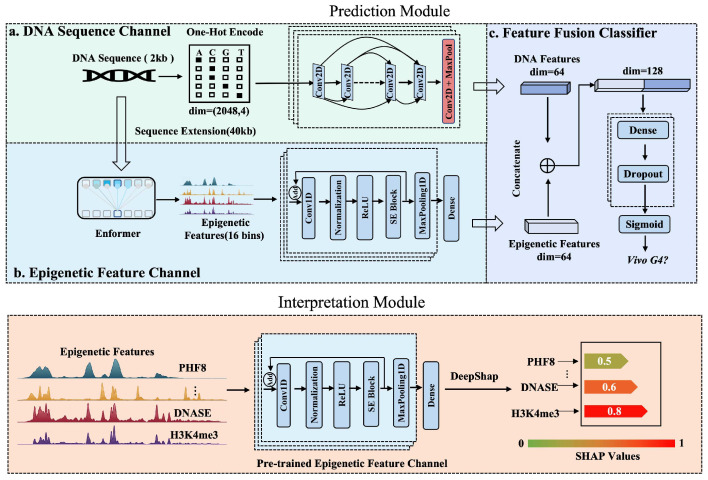
Schematic overview of the iDualG4 framework for in vivo G-quadruplex prediction and interpretation. The upper panel depicts the Prediction Module, which includes three main components: (**a**) the DNA Sequence Channel, (**b**) the Epigenetic Feature Channel, and (**c**) the Feature Fusion Classifier. The lower panel shows the Interpretation Module, in which DeepSHAP is used to quantify the contributions of inferred epigenetic features to the final prediction.

These limitations constitute key obstacles to moving current in vivo G4 predictions toward clinical and large-scale applications. Addressing these challenges, we propose iDualG4, an interpretable dual-channel deep learning framework designed to integrate intrinsic DNA sequence features with cell-specific epigenetic information relying solely on DNA sequence inputs (Figure 1). The model comprises a local sequence channel and an epigenetic proxy channel. Importantly, the term “epigenetic proxy” is used here in a specific sense. iDualG4 does not require newly measured, target-cell-specific epigenomic assays during inference; instead, it leverages Enformer as a pretrained sequence-to-function prior to infer regulatory context directly from DNA sequence. Because Enformer itself was trained on large-scale public genomic and epigenomic tracks, our approach does not eliminate all dependence on experimental epigenomic knowledge. Rather, it shifts this dependence from inference-time acquisition of matched assays to reusable pretraining-time knowledge. In this sense, the primary novelty of iDualG4 lies not merely in combining local and long-range sequence information, but in enabling interpretable in vivo G4 prediction under assay-limited settings by replacing matched epigenomic inputs with sequence-derived proxy features.The latter leverages sequence-to-function Transformer models, such as Enformer, to infer critical epigenetics-related signals (e.g., chromatin accessibility and H3K4me3) covering an approximately 40 kb region directly from raw sequences. By fusing these inferred signals with local sequence representations, iDualG4 models both local motifs and the long-range regulatory background simultaneously without requiring additional cell-specific sequencing data. Furthermore, we incorporate DeepSHAP to construct an interpretability framework, quantifying the relative contributions of different epigenetic signals to G4 formation and revealing potential synergistic roles of H3K4me3 and specific transcription factors. Overall, iDualG4 provides a novel computational scheme for high-precision prediction of in vivo G4s in scenarios lacking epigenomic data and offers a new perspective for systematically unraveling how sequence and the epigenetic environment jointly shape the G4 landscape.

## 2. Materialsand Methods

### 2.1. Data Sources and Preprocessing

To construct a comprehensive multi-cell-line binary classification dataset for the identification of in vivo G4 structures, we retrieved human G4-related high-throughput sequencing data—including ChIP-seq, CUT&Tag, and G4-seq—from the Gene Expression Omnibus (GEO) database. All datasets were subsequently unified under a standard reference genome coordinate system to ensure compatibility. In this study, positive samples were rigorously defined as G4 peaks captured by in vivo experiments, representing sequences that stably fold within the physiological chromatin context of living cells. Conversely, Putative Quadruplex Sequences (PQSs) derived from in vitro G4-seq experiments served as the source for negative samples. Specifically, positive samples corresponded to sequences capable of forming stable G4 structures in a cell-specific manner in vivo.

We utilized processed peak data provided by the original authors for three distinct cell lines: K562 (human chronic myelogenous leukemia) G4 ChIP-seq data (GSE107690) [19]; HepG2 (human liver cancer) G4 ChIP-seq data (GSE145090) [20]; and MCF7 (human breast cancer) G4 CUT&Tag data (GSE181373) [21]. The negative sample set was derived from genome-wide human in vitro G4-seq data (GSE110582) [22], which catalogues candidate sequences (PQS) possessing the physicochemical potential to form G4 structures. To address potential discrepancies arising from discordant reference genome versions across different studies, all raw coordinate data were standardized to the human reference genome assembly hg38 using the UCSC LiftOver tool [23]. Subsequently, all G4 sequence intervals were extended to cover a region of 1024 bp upstream and downstream from their respective center points.

To ensure high data fidelity, we performed a rigorous redundancy removal process. For the negative samples, we acknowledged that in vitro PQSs do not strictly equate to stably folded in vivo G4 structures. Therefore, we excluded any sites from the G4-seq PQS candidate set that overlapped with positive peaks identified in the K562, HepG2, or MCF7 cell lines. The specific exclusion criterion was defined as any overlap of ≥1 bp between a PQS and a positive peak. This filtration step yielded a refined negative set representing “sequences capable of forming G4 in vitro but unobserved in vivo”, thereby effectively reducing label noise and enhancing the discriminative power of the binary classification task.

These negative samples should therefore be interpreted as operational negatives rather than absolute non-G4 sequences: they represent loci with intrinsic in vitro G4-forming potential that were not observed as stable in vivo G4s under the current cell lines and experimental conditions. This definition inevitably introduces some label uncertainty, but it is biologically meaningful for the present task because it forces the model to distinguish intrinsic sequence potential from context-constrained stable in vivo folding.

For positive samples within each individual cell line, we screened for overlaps among in vivo G4 intervals; any entry exhibiting an overlap exceeding 10% of its own length was removed to eliminate redundant in vivo G4 records. To accommodate the input specifications of the Enformer model and to capture larger-scale regulatory contexts, we constructed long-range sequence windows of approximately 40 kb, anchored at the same center points. These extended sequences were input into the pretrained Enformer model [12] to generate genomic track predictions for the corresponding regions. The statistics of the processed dataset are summarized in Table 1.

### 2.2. iDualG4 Model Architecture

The framework comprises four core components: the DNA Sequence Channel, the Epigenetic Feature Channel, the Feature Fusion Classifier, and the Interpretation Module.

#### 2.2.1. DNA Sequence Channel

This module is designed to extract sequence-specific motifs associated with G4 formation to generate dense feature embeddings (Figure 1a). DNA sequences of 2048 bp were initially converted via one-hot encoding into matrices of dimension (2048, 4), explicitly representing base identity and sequential order. In terms of network architecture, the module first employed a 2D convolutional layer for local pattern extraction, configured with a kernel size of 4×4 and 128 filters, utilizing ReLU as the non-linear activation function to enhance feature expression. Subsequently, three Dense blocks were introduced to facilitate multi-scale feature learning. These blocks promote gradient propagation and feature reuse through concatenation, and are interleaved with MaxPool layers to progressively compress sequence length while retaining discriminative responses. To mitigate overfitting, Dropout regularization was applied after convolutional and dense blocks with rates of 0.2 (within dense blocks), 0.3 (after convolutional blocks), and 0.5 before the final fully connected layer. Finally, the convolutional features were flattened and projected into a low-dimensional latent representation vector via fully connected layers. The output vector dimension *d* was set to 64, achieved through two fully connected layers containing 256 and 64 neurons, respectively.

#### 2.2.2. Epigenetic Feature Channel

This module leverages the pretrained Enformer [12] model to capture the cell-specific chromatin environment. By fusing Convolutional Neural Networks (CNNs) and Transformers, Enformer processes long sequences and captures feature interactions across a broad receptive field via the global attention mechanism inherent to Transformer layers (Figure 1b). A 40 kb DNA sequence was input into the Enformer model, which generated 5313 genomic track features (including histone modifications, transcription factor binding profiles, and chromatin accessibility). For cell-specific prediction, these tracks were filtered using keywords (“K562”, “HepG2”, “MCF7”) to isolate epigenetic feature subsets relevant to each cell line. Since Enformer operates with a window size of 128 bp, we cropped the central 16 spatial bins (16×128bp=2048bp) to serve as the aligned input for this channel. In this study, Enformer is used as a pretrained sequence-to-function model that maps long DNA sequence context to genomic track predictions. Accordingly, the resulting features are treated as sequence-derived epigenetic proxies rather than direct experimental measurements from the target sample.

In the network design, the model first stacks two 1D convolutional layers (Conv1D) to extract local dependencies between different epigenetic marks. Each layer utilizes a kernel size of 5 and 256 filters, followed immediately by Batch Normalization to maintain training stability. To further enhance feature discrimination, a Squeeze-and-Excitation (SE) module—a channel-wise attention mechanism—was introduced with a reduction ratio of 16. This module adaptively recalibrates channel-wise feature responses by explicitly modeling interdependencies between channels, thereby effectively amplifying informative features while suppressing irrelevant noise. Furthermore, the model incorporates two consecutive residual convolutional blocks, each containing a convolution, an SE module, and a skip connection (Add), combined with MaxPool (pooling window and stride of 2) to progressively reduce dimensionality. The processed feature stream was finally mapped through three fully connected layers (Dense) with 256, 128, and 64 neurons, respectively. A Dropout rate of 0.3 was introduced before the output to prevent overfitting, resulting in a compressed 64-dimensional vector serving as the final representation of the cell-specific epigenetic environment.

#### 2.2.3. Feature Fusion Classifier

The module aims to deeply integrate the high-dimensional features extracted from the dual channels to perform the final classification task (Figure 1c). In the feature fusion stage, to maximally preserve the original feature space of both modalities, the feature vectors output by the DNA sequence channel and the epigenetic feature channel were concatenated to construct a unified joint feature vector. This vector was then processed by a classifier network composed of three fully connected layers (Dense). The first two hidden layers contained 128 and 64 neurons, respectively, and both utilized ReLU activation functions. Dropout regularization was introduced between fully connected layers with a uniform rate of 0.3 to prevent overfitting and improve generalization capabilities. Finally, the output layer, containing a single neuron, utilized a Sigmoid activation function to predict the probability of in vivo G4 formation.

#### 2.2.4. Interpretation Module

To dissect the specific contributions of epigenetic features to model predictions, we employed the DeepSHAP algorithm [24]. DeepSHAP integrates the backpropagation rules of DeepLIFT [25] with Shapley value theory [26,27,28]. This method utilizes the chain rule of multipliers defined by DeepLIFT to compare the difference between the current activation state of a neuron and a reference state (set as the average value of the feature across all samples). This mechanism allows importance signals to backpropagate through the neural network analogous to gradients. Building on this, DeepSHAP overcomes the limitations of traditional weight analysis by evaluating the marginal contribution of features across different subsets, calculating the SHAP value for each input feature (e.g., H3K4me3, DNase). Through these operations, DeepSHAP quantifies the positive or negative impact of specific epigenetic markers on the model’s prediction results.

### 2.3. Model Training and Evaluation

To establish the computational environment for model development and training, all experiments were conducted on an Ubuntu operating system deployed on a PowerEdge-T640 server, equipped with three NVIDIA GeForce RTX 3090 GPUs (24 GB VRAM each). The software framework was configured utilizing Python 3.8.16, CUDA 12.2, and the TensorFlow-GPU 2.5.0 deep learning framework. Additionally, the original version of the pretrained Enformer model released by DeepMind was utilized for epigenetic feature extraction.

The study employed a stratified 5-fold cross-validation scheme to partition the dataset, ensuring that the ratio of positive to negative samples in both training and testing sets remained consistent with the original data distribution. Binary Cross-Entropy was selected as the loss function, and the Adam optimizer with an initial learning rate of 0.0001 was used for parameter updates. To achieve refined training, a ReduceLROnPlateau learning rate scheduler was introduced to monitor validation loss in real-time; if the loss did not decrease for 3 consecutive epochs, the current learning rate was automatically decayed to 20% of its value (with a minimum lower bound of $1\times 10^{-6}$). Additionally, an Early Stopping strategy was employed to effectively prevent model overfitting. Specifically, we monitored the validation loss as the primary metric, setting the patience parameter to 10 epochs. Furthermore, a model checkpointing strategy was strictly implemented to ensure that the final model utilized for evaluation on the test set was restored to the exact weight configuration that achieved the lowest validation loss during the training phase. For model validation, Accuracy, Precision, Recall, F1-Score, Area Under the ROC Curve (AUC), and Area Under the PR Curve (AUPR) were utilized as comprehensive metrics to evaluate model performance.

## 3. Results

### 3.1. Comparative Experiments

To rigorously evaluate the predictive performance of iDualG4 in the in vivo G4 identification task, we benchmarked it against three representative methods across K562, HepG2, and MCF7 cell line datasets: XGBoost [8] (sequence-based); G4Beacon [9] (ATAC-seq integrated); and epiG4NN [10] (epigenetic fusion deep learning). All models were assessed using stratified 5-fold cross-validation, with results reported as mean ± standard deviation (Table 2).

Given the severe class imbalance inherent to this task—where negative samples significantly outnumber positives—the Area Under the Precision-Recall Curve (AUPR) provides a more robust measure of model effectiveness in retrieving rare positive events than standard accuracy metrics. Consequently, while we report overall metrics (Accuracy, AUC-ROC), this study prioritized sensitivity-focused metrics, including AUPR, F1-Score, Precision, and Recall (Table 2).

**Figure 2:**
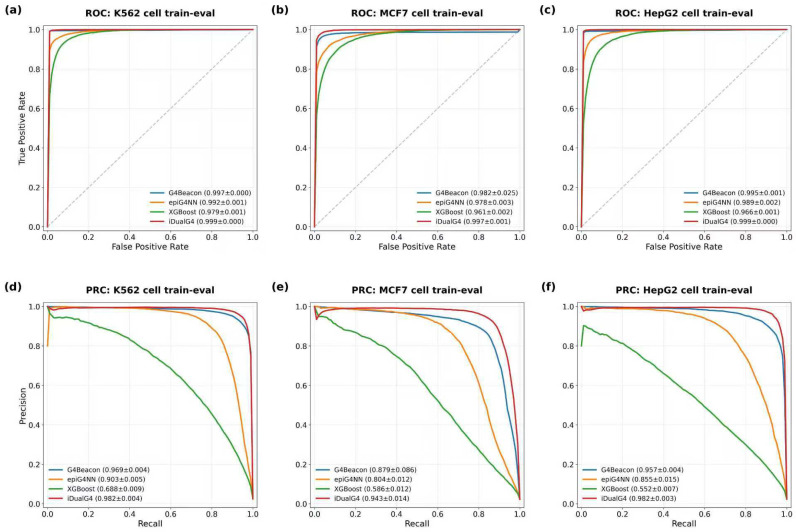
Performance assessment on independent test sets across three cell lines. ROC curves (**a**–**c**) and Precision-Recall Curves (PRC) (**d**–**f**) illustrate model accuracy. In the ROC plots, the gray dotted diagonal line denotes the random-classifier baseline (AUC = 0.5). Given the class imbalance inherent in in vivo G4 prediction, PRCs provide a more robust performance metric than ROCs. Panels correspond to: (**a**,**d**) K562; (**b**,**e**) MCF7; and (**c**,**f**) HepG2.

As detailed in Table 2, iDualG4 achieved the highest AUPR across all three cell lines. Notably, it outperformed the second-best method, G4Beacon, with AUPR improvements ranging from 0.004 in K562 to 0.063 in MCF7, demonstrating its superior capability in retrieving true positive G4s under imbalanced conditions. Beyond AUPR, iDualG4 also secured the highest F1-Scores (K562: 0.947, HepG2: 0.941, MCF7: 0.891), indicating a superior equilibrium between precision and recall. Figure 2 illustrates the ROC and PR curves; notably, the PR curves of iDualG4 sustain higher precision across a broader recall range. This larger area under the PR curve aligns with the quantitative metrics in Table 2, confirming that iDualG4 effectively suppresses false positives while enhancing detection sensitivity under imbalanced conditions.

In addition, the magnitude of improvement was not uniform across cell lines. The gain over the second-best baseline was relatively modest in K562 but became more pronounced in HepG2 and especially in MCF7. This pattern suggests that the benefit of iDualG4 is not merely due to stronger local motif recognition, but also reflects its ability to incorporate broader regulatory context when single-source information is insufficient. In particular, the larger AUPR margin in MCF7 indicates that prediction in this cell line may be more heterogeneous and less well captured by sequence-only or single-marker baselines, making the integration of long-range inferred epigenetic context particularly valuable. Conversely, the smaller improvement in K562 may reflect a setting in which baseline models already capture a larger proportion of easily recoverable signals, leaving less room for additional gains.

### 3.2. Ablation Studies

To quantify the specific contribution of each iDualG4 component, we conducted ablation studies evaluating three configurations: (1) DNA Sequence Channel only; (2) Epigenetic Feature Channel only; and (3) the complete dual-channel iDualG4 model. Results are summarized in Table 3.

The complete dual-channel model consistently yielded the highest AUPR and F1-Scores across all cell lines (Table 3). In K562 cells, for example, the complete model’s AUPR (0.981) substantially outperformed the DNA-only configuration (+0.073) and marginally improved upon the Epigenetics-only setup (+0.006). Similar performance trends were consistently observed in both HepG2 and MCF7 cell lines, confirming the advantage of the dual-channel integration. These findings indicate that intrinsic sequence features and epigenetic context provide complementary rather than redundant information. Notably, the epigenetic-only configuration already achieved high standalone AUPR in K562 and HepG2, suggesting that stable in vivo G4 formation in these cell lines is strongly constrained by chromatin-related context beyond sequence-intrinsic folding potential. However, the full dual-channel model consistently improved upon the epigenetic-only setting, indicating that local sequence features still contribute additional discriminative information that refines the decision boundary. This complementary effect was particularly evident in MCF7, where the performance gap between the complete model and either single-channel configuration was larger than in K562 and HepG2. Methodologically, this pattern implies that the relative importance of sequence versus proxy epigenetic context is cell-line-dependent; biologically, it is consistent with the idea that in vivo G4 formation emerges from the interplay between intrinsic G-rich sequence propensity and cell-specific regulatory environment rather than from either factor alone.

Notably, the Epigenetics-only module demonstrated high standalone AUPR (0.975/0.966/0.894), suggesting that epigenetic proxy features are strong predictors of stable in vivo G4 sites. The further improvement by the complete model implies that local sequence features contribute additional discriminative power, refining the precision-recall trade-off. Consequently, the iDualG4 architecture is not merely a data concatenation but effectively models the synergistic biological process of “sequence thermodynamic drive” coupled with “epigenetic environmental regulation.” This bio-inspired fusion enables the distinction between potential motifs and bona fide G4 sites, achieving predictive precision superior to either single channel.

### 3.3. Comparative Evaluation of Enformer Predicted Features Versus Epigenetic Sequencing Data

To validate the efficacy of “Enformer-based epigenetic proxy features” against “single experimental epigenetic markers,” we constructed three fusion baselines: DNA + ATAC-seq, DNA + H3K4me3, and DNA + Enformer. We performed consistent evaluations across the three cell lines (Table 4), where ATAC-seq represents chromatin accessibility and H3K4me3 serves as a transcriptional activation marker. For the experimental baselines, ATAC-seq and H3K4me3 sequencing data were sourced from ENCODE. Specifically, ATAC-seq accessions were ENCFF357GNC (K562), ENCFF976UNK (MCF7), and ENCFF262URW (HepG2) [9]; H3K4me3 accessions were ENCFF734RYD, ENCFF078BWS, and ENCFF581DXP. Data preprocessing involved extracting signal intensity from high-resolution 1 bp windows covering 1024 bp flanking the G4 sequence center, generating 1D signal vectors (length 2048) strictly aligned with the DNA sequence.

To ensure fair comparison, the dual-channel architecture was maintained: DNA inputs remained identical, while experimental ATAC-seq or H3K4me3 vectors replaced the Enformer matrix in the Epigenetic Feature Channel. All comparisons utilized the same 5-fold cross-validation strategy. Results demonstrate that the DNA+Enformer configuration achieved the highest AUPR (K562: 0.981, MCF7: 0.942, HepG2: 0.982). Compared with DNA + ATAC, AUPR improved by 0.015, 0.054, and 0.021, respectively; compared with DNA + H3K4me3, improvements were 0.040, 0.084, and 0.041 (Table 4). This confirms that within this experimental setting, the multi-track epigenetic proxy features predicted by Enformer provide substantially greater information gain than fusion approaches relying on single epigenetic markers (ATAC or H3K4me3).

A likely explanation for this advantage is that Enformer-derived proxy features are not limited to a single biochemical signal, but instead encode a broader regulatory context by jointly modeling multiple genomic tracks over an extended sequence window. In contrast, ATAC-seq and H3K4me3 each represent only one facet of chromatin state—accessibility or active histone marking, respectively. The stronger performance of DNA+Enformer therefore suggests that stable in vivo G4 formation is better characterized by a composite regulatory landscape than by any single experimental marker alone. This interpretation is further supported by the fact that the largest improvement was observed in MCF7, where reliance on one epigenetic mark appears less sufficient and broader contextual information provides greater added value.

### 3.4. DeepSHAP Reveals Sequence and Epigenetic Drivers of G4 Formation

We employed interpretability analysis to investigate the decision-making basis of the model and dissect regulatory associations between multi-dimensional epigenetic features and in vivo G4 formation. Given the heterogeneity in biological relevance across Enformer’s cell-type-specific features, we utilized DeepSHAP to identify drivers of G4 formation. This involved calculating Shapley Additive Explanations (SHAP) importance scores for input nodes to quantitatively assess feature contribution weights. Technically, we adopted the DeepSHAP framework based on Integrated Gradients to quantify Enformer feature importance for all in vivo G4 positive samples in K562, HepG2, and MCF7 lines.

**Figure 3:**
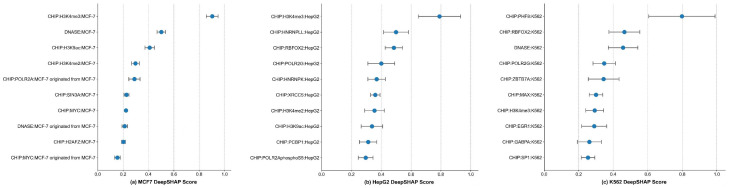
Epigenetic feature importance analysis. The relative contribution of epigenetic features to model predictions is visualized for (**a**) MCF7, (**b**) HepG2, and (**c**) K562 cell lines.

Analysis of the top-ranked features revealed several recurrent patterns that are biologically plausible and consistent with prior studies (Figure 3). First, H3K4me3 was consistently assigned high importance across all three cell lines, suggesting that active chromatin-associated signals are strongly used by the model when identifying stable in vivo G4s. Likewise, DNase-related features received high importance in multiple settings, indicating that chromatin accessibility is an important component of the predictive context captured by the model. In addition, several transcription factor-related features showed cell-line-specific enrichment among the top-ranked signals, such as PHF8, MAX, EGR1, and ZBTB7A in K562; HNRNPK, HNRNPLL, XRCC5, and PCBP1 in HepG2; and POLR2A, SIN3A, MYC, H2AFZ, and MAZ in MCF7. These observations suggest that, beyond broadly shared chromatin features, the model also captures cell-context-dependent regulatory patterns.

To evaluate the robustness of these attributions, we further examined the consistency of the top 10 DeepSHAP-ranked features across the five cross-validation folds. Several features were repeatedly recovered across all folds (Table S1), including H3K4me2 and H3K4me3 in all three cell lines, supporting their role as stable model-prioritized predictors. In contrast, other high-ranking features were cell-line-specific, indicating that the regulatory contexts associated with in vivo G4 formation may differ across cellular backgrounds (Table S2). Overall, these results should be interpreted as model-derived hypotheses and attribution patterns, rather than direct causal evidence of biological mechanism.

### 3.5. Parameter Sensitivity Analysis

**Figure 4:**
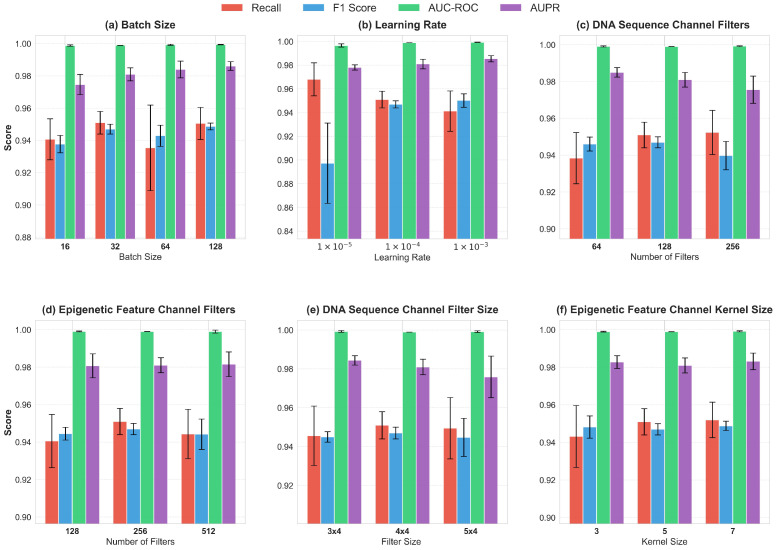
Hyperparameter sensitivity analysis. Model performance is evaluated using Recall (Red), F1-Score (Blue), AUC-ROC (Green), and AUPR (Purple). Black error bars denote the standard deviation from independent experiments. Panels display the effects of: (**a**) batch size; (**b**) learning rate; (**c**,**d**) number of filters in the DNA sequence and epigenetic feature Channel, respectively; (**e**) filter size in the DNA sequence channel; and (**f**) kernel size in the epigenetic feature channel.

To comprehensively evaluate iDualG4’s robustness and validate hyperparameter selection, we conducted a parameter sensitivity analysis on the K562 dataset using the One-Factor-at-a-Time (OFAT) method—an experimental design that involves changing one parameter at a time while holding all others constant to assess individual impacts (Figure 4). We assessed the impact of training hyperparameters (learning rate, Batch Size) and architecture parameters (filter count, kernel size) on Recall, F1-Score, AUC-ROC, and AUPR.

Experimental results indicate that reducing the learning rate to $1\times 10^{-5}$ caused a significant drop in F1-Score (to 0.8973) and increased standard deviation, suggesting hindered convergence or local minima entrapment; conversely, the baseline rate of $1\times 10^{-4}$ ensured high accuracy and stability. While increasing Batch Size to 128 yielded a marginal AUPR improvement, the baseline of 32 offered more balanced performance across all metrics. Regarding architecture, setting the DNA Sequence Channel filter count to 128 optimized both F1 and Recall, whereas varying Epigenetic Feature Channel filters (128–512) had negligible impact. Furthermore, kernel size analysis indicated that a 4×4 kernel for the DNA Sequence Channel and a size 5 kernel for the Epigenetic Feature Channel achieved the optimal balance between capturing local motifs and long-range dependencies.

### 3.6. Robustness Against Label Noise

While using in vitro PQSs as negatives provides a large-scale background, it may introduce potential label noise, as some loci with G4-forming potential might still adopt stable G4 conformations under specific biological contexts not captured in current in vivo datasets. To explicitly examine the impact of this label noise on model learning, we performed an additional robustness analysis using a stricter negative-sample definition. Specifically, we extended each positive in vivo G4 peak by ±1 kb and strictly excluded any PQSs overlapping with these extended regions to construct a refined negative set.

Using the same 5-fold cross-validation protocol, we retrained and evaluated both iDualG4 and the second-best baseline, G4Beacon. Under this stricter setting, iDualG4 remained consistently superior to G4Beacon across all three cell lines, maintaining high predictive performance (e.g., AUPR of 0.9822±0.0026 on HepG2 and 0.9768±0.0072 on K562). The detailed performance metrics are provided in Supplementary Table S3. This ranking stability confirms that our main conclusions are robust and are not driven by obvious boundary-related label noise.

## 4. Discussion

The findings of this study computationally support the “sequence + environment” dual-drive hypothesis, reflecting the underlying biology of in vivo G4 formation: G4 folding is not a static property but a dynamic equilibrium tightly modulated by the local chromatin microenvironment [4]. While intrinsic DNA motifs provide the thermodynamic basis and folding potential [29], they are insufficient to guarantee stable existence within the nucleus. The cell-specific epigenetic environment (e.g., chromatin accessibility, histone modifications) plays a critical role in regulating the competition between G4 and double-stranded DNA, determining whether thermodynamic potential translates into structural reality [6]. Consequently, our observation that Enformer-predicted proxies contribute more to model performance than single-source experimental data (e.g., ATAC-seq) underscores the necessity of integrating multi-omics prior knowledge to capture this complex regulatory process.

Relative to state-of-the-art methods, iDualG4 exhibits enhanced robustness on datasets with extreme class imbalance, significantly mitigating false positive rates. Distinct from studies limited to black-box prediction, we leveraged DeepSHAP for interpretability. This analysis not only confirmed the known positive correlation between chromatin accessibility (DNase) and G4 formation but also identified a specific association between the histone demethylase PHF8 and G4s in K562 cells—a regulatory relationship underexplored in previous predictive models [30]. Based on our interpretability results, we propose that G4 formation extends beyond a passive thermodynamic process; it likely undergoes refined regulation via the active recruitment or stabilization by specific transcription factors, such as PHF8. These factors may establish a “permissive microenvironment” for G4 folding by remodeling local chromatin conformation.

Furthermore, a critical aspect of practical model utility is its generalizability to unknown cell types or clinical samples, where matched epigenomic profiling is frequently absent. To rigorously evaluate this, we conducted a “leave-one-cell-line-out” cross-cell-line generalization analysis. In this zero-shot transfer setup, models were iteratively trained on two cell lines and tested on a strictly unseen third cell line. To preclude data leakage, sequences in the training set that shared overlapping genomic coordinates with the hold-out test set were stringently excluded. While absolute performance metrics naturally declined compared with intra-cell-line cross-validation—reflecting the profound heterogeneity of cell-type-specific epigenetic landscapes—iDualG4 consistently outperformed the baseline G4Beacon across all scenarios. Specifically, for AUPR, iDualG4 achieved 0.5648 (vs. G4Beacon’s 0.5355) when predicting on unseen K562 cells, 0.6778 (vs. 0.5886) on HepG2, and 0.7318 (vs. 0.6425) on MCF7. Parallel improvements were maintained across Precision, Recall, and F1-Scores. The detailed cross-cell-line generalization metrics are provided in Supplementary Table S4. The results show that cross-cell-line transfer is substantially more challenging than within-cell-line cross-validation, as expected given the heterogeneity of cell-type-specific chromatin landscapes. Nevertheless, iDualG4 retained a modest but consistent advantage over G4Beacon in all three leave-one-cell-line-out settings, suggesting that the proposed framework has some transfer potential while also highlighting the need for further validation in more diverse cell types and clinical samples.

Despite these advances, limitations remain. A further conceptual nuance should be emphasized. Although iDualG4 does not require matched cell-specific epigenomic assays during inference, the Enformer module used in our framework is itself pretrained on large-scale public genomic and epigenomic datasets. Therefore, our approach should not be interpreted as being completely independent of experimental epigenomic knowledge. Rather, it transfers such knowledge from explicit assay input at prediction time to an implicit sequence-to-function prior learned during pretraining. This design is particularly advantageous in settings where matched epigenomic profiles are unavailable, but it also means that the quality and scope of the inferred proxy features remain bounded by the representational capacity of the pretrained Enformer model. A further limitation is that the current framework explicitly models local sequence determinants and Enformer-derived epigenetic proxy features, but does not account for additional chemical or structural modulators of G4 folding. In particular, recent biophysical studies have shown that small-molecule ligands can alter the conformational equilibria, intermediate states, and structural preferences of G-rich sequences and promoter-associated G4s. Therefore, while iDualG4 captures two major determinants of stable in vivo G4 formation—sequence potential and regulatory chromatin context—it does not yet model ligand-mediated structural plasticity, which represents an important direction for future work [31,32,33].

## 5. Conclusions

In this study, we presented and validated iDualG4, a dual-channel framework for the identification of in vivo G4 structures. Experimental evaluations across three human cancer cell lines (K562, HepG2, and MCF7) demonstrate that iDualG4 achieves high-precision prediction. Notably, in terms of AUPR, the model consistently outperforms baselines relying solely on sequence motifs or single epigenetic features. By integrating Enformer, the model enables “end-to-end” prediction from sequence alone, bypassing the requirement for cell-specific experimental inputs and thereby broadening its utility.

Future iterations could implement Attention Mechanisms to selectively filter for high-confidence epigenetic features or integrate single-cell sequencing data for higher-resolution calibration. Furthermore, future work will prioritize: (1) experimentally validating the biological functions of novel G4 regulators (e.g., PHF8) prioritized by DeepSHAP; and (2) incorporating RNA-seq data to investigate the feedback mechanisms of transcriptional activity on DNA G4 stability.

## Figures and Tables

**Table 1:** Statistics of the processed datasets for K562, MCF7, and HepG2.

Cell Line	Label (Data Source)	Count
K562	Positive (in vivo G4, G4 ChIP-seq)	7957
	Negative (PQS, G4-seq)	319,694
MCF7	Positive (in vivo G4, G4 CUT&Tag)	7319
	Negative (PQS, G4-seq)	320,427
HepG2	Positive (in vivo G4, G4 ChIP-seq)	7580
	Negative (PQS, G4-seq)	319,836

**Table 2:** Benchmarking iDualG4 against existing methods on K562, HepG2, and MCF7 cell lines.

Dataset	Models	Precision	Recall	F1-Score	Accuracy	AUC-ROC	AUPR
K562	G4Beacon	0.918±0.006	0.945±0.006	0.931±0.005	0.996±0.001	0.998±0.001	0.977±0.003
	epiG4NN	0.835±0.014	0.843±0.012	0.839±0.006	0.991±0.001	0.991±0.001	0.903±0.004
	XGBoost	0.764±0.007	0.502±0.010	0.606±0.009	0.983±0.001	0.978±0.001	0.688±0.008
	**iDualG4**	0.943±0.007	0.951±0.007	0.947±0.003	0.997±0.000	0.999±0.000	0.981±0.004
HepG2	G4Beacon	0.886±0.008	0.923±0.006	0.904±0.007	0.995±0.001	0.996±0.001	0.961±0.003
	epiG4NN	0.779±0.028	0.778±0.028	0.777±0.010	0.989±0.001	0.989±0.002	0.854±0.015
	XGBoost	0.692±0.009	0.361±0.005	0.474±0.005	0.981±0.001	0.965±0.001	0.551±0.006
	**iDualG4**	0.928±0.027	0.954±0.007	0.941±0.011	0.997±0.001	0.999±0.000	0.982±0.002
MCF7	G4Beacon	0.853±0.050	0.848±0.007	0.849±0.024	0.993±0.001	0.982±0.025	0.879±0.085
	epiG4NN	0.763±0.024	0.735±0.021	0.748±0.008	0.988±0.001	0.978±0.002	0.804±0.011
	XGBoost	0.742±0.010	0.405±0.010	0.524±0.010	0.983±0.001	0.960±0.002	0.585±0.011
	**iDualG4**	0.905±0.018	0.878±0.024	0.891±0.008	0.995±0.000	0.997±0.001	0.942±0.011

*Note:* Bold values indicate the best performance for each metric within the same dataset.

**Table 3:** Ablation analysis of the iDualG4 model across K562, MCF7, and HepG2 cell lines.

Dataset	Ablated Module	Precision	Recall	F1-Score	Accuracy	AUC-ROC	AUPR
K562	DNA Sequence Channel	0.876±0.027	0.808±0.053	0.839±0.018	0.992±0.001	0.995±0.001	0.908±0.017
	Epigenetic Feature Channel	0.927±0.008	0.932±0.008	0.930±0.002	0.996±0.001	0.998±0.001	0.975±0.001
	**iDualG4**	0.943±0.007	0.951±0.007	0.947±0.003	0.997±0.000	0.999±0.000	0.981±0.004
MCF7	DNA Sequence Channel	0.881±0.024	0.666±0.035	0.758±0.021	0.990±0.001	0.986±0.003	0.826±0.020
	Epigenetic Feature Channel	0.884±0.025	0.789±0.044	0.832±0.015	0.992±0.001	0.988±0.002	0.894±0.011
	**iDualG4**	0.905±0.018	0.878±0.024	0.891±0.008	0.995±0.000	0.997±0.001	0.942±0.011
HepG2	DNA Sequence Channel	0.826±0.046	0.723±0.082	0.765±0.034	0.989±0.001	0.992±0.001	0.852±0.014
	Epigenetic Feature Channel	0.911±0.014	0.919±0.014	0.915±0.004	0.995±0.001	0.998±0.001	0.966±0.003
	**iDualG4**	0.928±0.027	0.954±0.007	0.941±0.011	0.997±0.001	0.999±0.000	0.982±0.002

*Note:* Bold values indicate the best performance for each metric within the same dataset.

**Table 4:** Comparison of model performance using Enformer-predicted features versus experimental epigenetic markers.

Dataset	Input Combination	Precision	Recall	F1-Score	Accuracy	AUC-ROC	AUPR
	DNA + ATAC	0.944±0.030	0.893±0.046	0.917±0.021	0.995±0.001	0.998±0.000	0.966±0.014
K562	DNA + H3K4me3	0.924±0.031	0.836±0.051	0.876±0.021	0.994±0.001	0.996±0.001	0.941±0.008
	**DNA + Enformer**	0.943±0.007	0.951±0.007	0.947±0.003	0.997±0.000	0.999±0.000	0.981±0.004
	DNA + ATAC	0.900±0.046	0.777±0.048	0.831±0.014	0.992±0.001	0.983±0.002	0.888±0.017
MCF7	DNA + H3K4me3	0.862±0.031	0.755±0.032	0.803±0.012	0.991±0.001	0.988±0.002	0.858±0.012
	**DNA + Enformer**	0.905±0.018	0.878±0.024	0.891±0.008	0.995±0.000	0.997±0.001	0.942±0.011
	DNA + ATAC	0.915±0.020	0.928±0.017	0.921±0.012	0.996±0.001	0.997±0.002	0.961±0.016
HepG2	DNA + H3K4me3	0.924±0.025	0.826±0.042	0.871±0.017	0.994±0.001	0.997±0.000	0.941±0.005
	**DNA + Enformer**	0.928±0.027	0.954±0.007	0.941±0.011	0.997±0.001	0.999±0.000	0.982±0.002

*Note:* Bold values indicate the best performance for each metric within the same dataset.

## Data Availability

The source code with this paper has been deposited in the GitHub repository, version v1.0.0: https://github.com/fukaluosi88-bit/iDualG4 ( accessed on 4 May 2026). All sequencing datasets analyzed in this study are publicly available. G4 profiling data were obtained from the NCBI Gene Expression Omnibus (GEO; https://www.ncbi.nlm.nih.gov/geo/ (accessed on 4 May 2025)), including G4 ChIP-seq for K562 (GSE107690) and HepG2 (GSE145090), G4 CUT&Tag for MCF7 (GSE181373), and in vitro G4-seq data (GSE110582). In addition, epigenomic profiles were downloaded from the ENCODE portal (https://www.encodeproject.org/ (accessed on 4 May 2025)). Specifically, ATAC-seq datasets for K562, MCF7, and HepG2 correspond to accessions ENCFF357GNC, ENCFF976UNK, and ENCFF262URW, respectively, and H3K4me3 datasets correspond to ENCFF734RYD, ENCFF078BWS, and ENCFF581DXP, respectively.

## Supplementary Materials

The following supporting information can be downloaded at: https://www.mdpi.com/article/10.3390/biom16050693/s1, Table S1: Frequency of top 10 epigenetic features identified by DeepSHAP across 5-fold cross-validation in K562, HepG2, and MCF7 cell lines; Table S2: Categorization of recurrent DeepSHAP-prioritized epigenetic features into cross-cell-line shared and cell-line-specific features; Table S3: Robustness analysis of iDualG4 and G4Beacon using a stricter negative-sample definition (excluding PQSs within ±1 kb of positive peaks); Table S4: Cross-cell-line generalization performance (leave-one-cell-line-out) for iDualG4 and G4Beacon.

## Abbreviations

Abbreviation	Full term	Brief meaning
G4	G-quadruplex	Four-stranded guanine-rich nucleic acid structure
PQS	Putative G4-forming sequence	Sequence with potential to form G4
DHS	DNase I hypersensitive site	Marker of chromatin accessibility
OFAT	One-factor-at-a-time	Hyperparameter sensitivity analysis strategy
SE block	Squeeze-and-excitation block	Channel attention module
SHAP	Shapley additive explanations	Feature attribution method
