# Supplementary material for: iDualG4: A Dual-Channel Deep Learning Framework for Predicting In Vivo G-Quadruplexes
Source: Biomolecules. 2026 May 7;16(5):693. doi: 10.3390/biom16050693 (PMC13204512; doi:10.3390/biom16050693)
Supplement: Supplementary file 1 [file biomolecules-16-00693-s001.zip › biomolecules-4248844-supplementary.pdf]

Supplementary Materials

iDualG4: A dual-channel deep learning framework for predicting in vivo G-quadruplexes

Haitao Li[1], Li Dong[2], Yue Jia[2], Chunhou Zheng[1] and Pijing Wei[2,*]

Key Laboratory of Intelligent Computing and Signal Processing, School of Artificial Intelligence, Anhui University, 111 Jiulong Road, Hefei 230601, China

Physical Science and Information Technology, Anhui University, 111 Jiulong Road, Hefei 230601, China

*Corresponding author:Pijing Wei. E-mail:weipj@ahu.edu.cn

Supplementary Table

Table S1. Frequency of top 10 epigenetic features identified by DeepSHAP across 5-fold cross-validation in K562, HepG2, and MCF7 cell lines.

Cell Line	Feature Name	Appearance Count (5-fold)
K562	CHIP:PHF8:K562	5
	CHIP:RBFOX2:K562	5
	DNASE:K562	5
	CHIP:POLR2G:K562	5
	CHIP:ZBTB7A:K562	5
	CHIP:MAX:K562	5
	CHIP:H3K4me3:K562	5
	CHIP:EGR1:K562	4
	CHIP:H3K4me2:K562	4
	CHIP:GABPA:K562	2
HepG2	CHIP:H3K4me3:HepG2	5
	CHIP:HNRNPLL:HepG2	5
	CHIP:RBFOX2:HepG2	5
	CHIP:POLR2G:HepG2	5
	CHIP:H3K4me2:HepG2	5
	CHIP:HNRNPK:HepG2	5
	CHIP:XRCC5:HepG2	5
	CHIP:PCBP1:HepG2	5
	CHIP:H3K9ac:HepG2	4
	CHIP:POLR2AphosphoS5:HepG2	2
MCF7	CHIP:H3K4me3:MCF-7	5
	DNASE:MCF-7	5
	CHIP:H3K9ac:MCF-7	5
	CHIP:POLR2A:MCF-7originated from MCF-7	5
	CHIP:H3K4me2:MCF-7	5
	CHIP:SIN3A:MCF-7	5
	CHIP:MYC:MCF-7	5
	DNASE:MCF-7originated from MCF-7	5
	CHIP:H2AFZ:MCF-7	5
	CHIP:MAZ:MCF-7	3

This table presents the top 10 epigenetic features associated with G-quadruplex formation across three cell lines: K562, HepG2, and MCF7. The data in this table was obtained by utilizing DeepSHAP to extract key signals, and the consistency of these features was statistically evaluated through a 5-fold cross-validation experiment to ensure the stability and reliability of the model's feature identification.

Table S2. Categorization of recurrent DeepSHAP-prioritized epigenetic features into cross-cell-line shared and cell-line-specific features.

Category	Cell Line Scope	Stable Core Epigenetic Features
Cross-cell-line share	K562，HepG2，MCF7	CHIP:H3K4me2, CHIP:H3K4me3
Cell-line-unique	K562	CHIP:PHF8, CHIP:MAX, CHIP:EGR1, CHIP:ZBTB7A
	HepG2	CHIP:HNRNPK, CHIP:HNRNPLL,CHIP:XRCC5, CHIP:PCBP1
	MCF7	CHIP:POLR2A, CHIP:SIN3A, CHIP:MYC,CHIP:H2AFZ, CHIP:MAZ

This table categorizes the stable core epigenetic features derived from the consistency analysis. It classifies these identified signals into cross-cell-line shared regulators and cell-line-unique regulators across K562, HepG2, and MCF7, illustrating both the conserved and context-specific epigenetic environments related to G4 formation.

Table S3. Robustness analysis of iDualG4 and G4Beacon using a stricter negative-sample definition(excluding PQSs within±1 kb of positive peaks).

Model	Cell Line	Precision	Recall	F1 Score	Accuracy	AUC-ROC	AUPR
**iDualG4**	**HepG2**	0.9460±0.0135	0.9387±0.0110	0.9421±0.0034	0.9972±0.0002	0.9994±0.0001	0.9822±0.0026
	**MCF7**	0.8835±0.0307	0.8791±0.0345	0.8802±0.0096	0.9945±0.0004	0.9962±0.0013	0.9363±0.0113
	**K562**	0.9503±0.0216	0.9164±0.0362	0.9323±0.0152	0.9967±0.0007	0.9990±0.0003	0.9768±0.0072
**G4Beacon**	**HepG2**	0.9310±0.0140	0.9237±0.0118	0.9273±0.0038	0.9952±0.0003	0.9964±0.0002	0.9672±0.0028
	**MCF7**	0.8435±0.0315	0.8391±0.0352	0.8413±0.0099	0.9965±0.0005	0.9950±0.0016	0.8963±0.0118
	**K562**	0.9353±0.0220	0.9014±0.0368	0.9180±0.0155	0.9967±0.0008	0.9980±0.0004	0.9618±0.0075

This table presents a robustness analysis comparing the iDualG4 model with the G4Beacon method. This evaluation is conducted by constructing stricter negative samples, specifically by excluding putative G-quadruplex sequences (PQSs) located within±1 kb of the positive peaks. The table details the performance of both models across three cell lines (HepG2, MCF7, and K562) using a comprehensive set of evaluation metrics, including Precision, Recall, F1-Score, Accuracy, AUC ROC, and AUPR.

Table S4. Cross-cell-line generalization performance (leave-one-cell-line-out) for iDualG4 and G4Beacon.

Model	Cross-Validation Setup	Precision	Recall	F1 Score	Accuracy	AUC-ROC	AUPR
**iDualG4**	MCF7-HepG2--->K562	0.6001	0.5998	0.6000	0.6000	0.5940	0.5648
	MCF7-K562--->HepG2	0.6369	0.6010	0.6184	0.6292	0.7682	0.6778
	K562-HepG2-->-MCF7	0.6002	0.5999	0.6000	0.6001	0.6225	0.7318
**G4Beacon**	MCF7-HepG2--->K562	0.5712	0.5685	0.5698	0.5705	0.5628	0.5355
	MCF7-K562--->HepG2	0.5452	0.5125	0.5283	0.5388	0.6795	0.5886
	K562-HepG2-->-MCF7	0.5115	0.5082	0.5098	0.5105	0.5318	0.6425

This table details the cross-cell-line generalization performance of the iDualG4 and G4Beacon models. To rigorously assess the models' ability to generalize to unseen

data, a leave-one-cell-line-out cross-validation strategy was employed. In this setup, the models were trained on data from two cell lines and subsequently evaluated on the third, independent cell line (e.g., training on MCF7 and HepG2, testing on K562). The table summarizes key evaluation metrics—including Precision, Recall, F1-Score, Accuracy, AUC ROC, and AUPR—facilitating a comprehensive comparison of the models' predictive robustness and transferability across diverse cellular environments.
